# The Harmony and Balance of the Facial Organs for a Natural Face Beauty: A Novel Perspective for Cosmetic/Aesthetic Interventions

**DOI:** 10.3390/medicina61060958

**Published:** 2025-05-22

**Authors:** Serdar Babacan, Mustafa Deniz

**Affiliations:** 1 Department of Anatomy, Medical Faculty, Bursa Uludag University, Bursa 16059, Türkiye; 2 Department of Anatomy, Medical Faculty, Harran University, Şanlıurfa 63250, Türkiye; denizmf@homail.com

**Keywords:** facial beauty, facial harmony, natural face beauty, cosmetic/aesthetic interventions

## Abstract

*Background and Objectives*: Facial beauty has attracted the attention of human societies for centuries, but there is not yet a common universal consensus on the perception of beauty. The first stage of facial analysis is a frontal examination of the face. Therefore, determining the morphometric characteristics of the face and facial organs will help to perceive the nuances that influence the aesthetics specific to each person. The aim of our study is to develop regression equations that will design personalized morphometric features for reconstructive and aesthetic applications that will adapt to each individual’s personal face and facial organs and incorporate cultural elements. *Materials and Methods*: The study was conducted with 100 volunteers, 50 males (mean age = 21.48 ± 1.54 years) and 50 females (mean age = 21.26 ± 0.66 years). We took facial photographs of the participants in the Frankfurt Horizontal plane so that measurements of the face and facial organs could be made on digital media. We measured forty parameters (eight for face, twelve for eyes, eight for nose, and twelve for lips). We used Image J (ver. 1.51) software for the measurements. We used SPSS Ver. 28.0 for the statistical analysis of the data. *Results*: As a result of the comparative statistical analysis, statistically significant (*p* < 0.05) differences were found between men and women in the F5—lower face height, E5—palpebral fissure height, E6—distance between the margin of the upper eyelid and the eyebrow, E8—distance between the midpoint of the eye and the edge of the lower eyelid, N3—alar width, and N5—nasal root angle variables. *Conclusions*: On the basis of the correlation analyses, linear regression equations were developed to estimate the ideal natural facial morphometry of men and women by the means of variables that were highly correlated with each other. The equations developed will estimate the optimum morphometric features of a person for natural harmony and balance between the face and facial organs in accordance with the individual’s population and gender. We believe that our study will guide medical professionals who perform cosmetic/aesthetic interventions and also inspire software or artificial intelligence applications related to facial or facial organ design.

## 1. Introduction

The face is the first identifying anatomical region perceived and evaluated by humans [1]. Therefore, facial beauty has attracted the attention of human societies for centuries, but there is not yet a common universal consensus on the perception of beauty [2]. It is not possible to explain beauty with a single element. Many elements of the human face are responsible for beauty. Fundamental components of aesthetic perception, such as harmony and symmetry, are largely predicated on the principle of proportion [3,4]. Proportion is a constant and parallel relationship with other structures, and harmony means an optimal proportional relationship [5]. The ancient Greeks also questioned the meaning of beauty and believed that the world was beautiful. They concluded that this beauty resulted from a certain order, harmony, measure, and proportion between the elements [6,7]. Although the perception of beauty can be objectively evaluated among different cultures and ethnic groups based on the neoclassical canon and the golden ratio, there is also a subjective perspective based on genetic, cultural, and environmental factors [7].

The basic elements of facial aesthetics and beauty include facial analysis based on symmetry and harmony [8,9,10]. The principles of aesthetic design that represent how to put things together to create and communicate beauty are balance, proportion, simplicity, unity, symmetry, and harmony [11]. In general terms, there is no unique and identifiable prototype of a beautiful face. The attractiveness of a face lies in its overall harmony and integration with the rest of the face [12]. Each culture has its own standards of facial beauty. Therefore, there is no universal standard for facial measurements. For example, in one region, the representative elements of beauty may be an oval or round face, arched eyebrows, a small nose, a pointed chin, large eyes, prominent cheeks, and full lips, while another region may have different standards [13].

The harmony and balance of facial organs involve a complex interplay of features that contribute to aesthetic appeal, professional development, social interaction, and functional effectiveness in self-confidence and self-esteem. This concept is based on the principles of symmetry, proportion, and dynamic relationships between facial structures. Across cultures and across ages, perceptions of beauty have often emphasized the importance of a harmonious image in which each element, from the eyes to the nose, lips, and chin, works together seamlessly. Beyond mere aesthetics, facial harmony plays a crucial role in expression and communication by reflecting emotions and personality [14,15,16,17,18].

The first stage of facial analysis is a frontal examination of the face [19]. Therefore, determining the morphometric characteristics of the face and facial organs will help to perceive the nuances that affect individual aesthetics [4,20,21]. Aesthetic–surgical perspectives are important for improving the morphometry, geometry, and proportions of the face and facial organs [22,23].

Today, thanks to advances in technology, it is possible to produce attractive and desirable results in facial analysis. However, direct facial analysis with a careful clinical examination remains the primary way of examining the face because it is cost-effective, easily accessible, and feasible, providing more natural and predictable results, enabling the development of skills to distinguish individual features and a plan for facial harmony based on facial proportions in terms of personalized planning [4].

We believe that an approach that focuses more on balance, harmony, and the preservation of cultural facial features specific to the society in which the person lives should be adopted in beauty perception and aesthetic applications. Therefore, the aim of our study is to develop regression equations that will design individual-specific morphometric features for reconstructive and aesthetic applications that will adapt to the individual’s personal face and facial organs and incorporate cultural elements.

## 2. Materials and Methods

**Figure 1:**
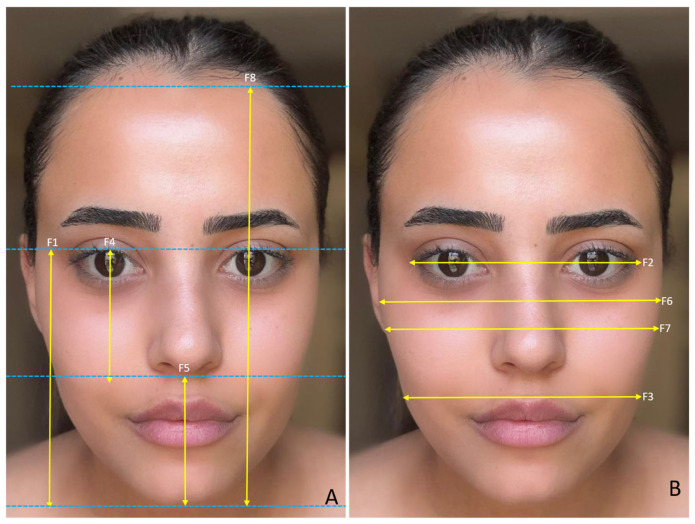
(**A**,**B**) Measured variables related to the face. F1—facial height (distance between nasion and menton); F2—upper face width (distance between external corner of the right eye and external corner of the left eye); F3—lower face width (bigonial width); F4—upper face height (distance between nasion and subnasale); F5—lower face height (distance between nasion and menton); F6—inter-tragus width; F7—facial width (bizygomatic width); and F8—total facial height.

**Figure 2:**
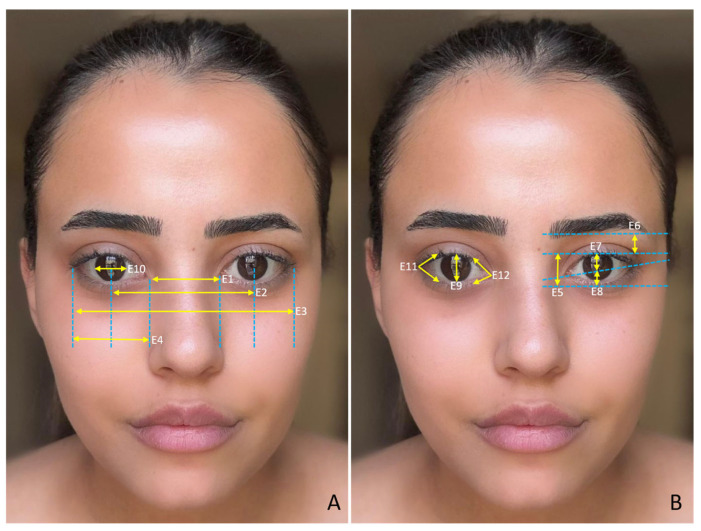
(**A**,**B**) Measured variables related to the eye. E1—internal intercantal distance; E2—interpupillary distance; E3—external intercantal distance; E4—palpebral fissure length (distance between medial and lateral canthus (eye width)); E5—palpebral fissure height (distance between upper and lower eyelids); E6—distance between the margin of the upper eyelid and the eyebrow; E7—distance between the midpoint of the eye and the edge of the upper eyelid; E8—distance between the midpoint of the eye and the edge of the lower eyelid; E9—vertical diameter of the iris (apparent); E10—horizontal diameter of the iris (apparent); E11—lateral canthus angle; and E12—medial canthus angle.

**Figure 3:**
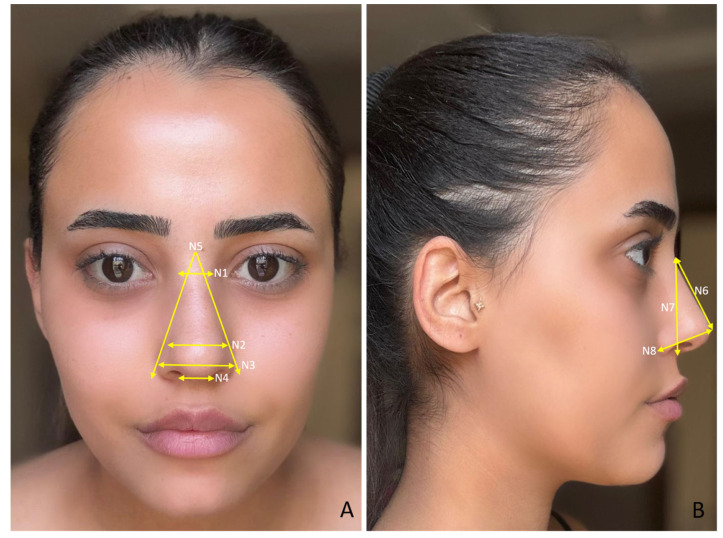
(**A**,**B**) Measured variables related to the nose. N1—nasal root width; N2—nasal dorsum width; N3—alar width; N4—subalar width; N5—nasal root angle; N6—nasal length; N7—nasal height; and N8—nasal tip projection.

**Figure 4:**
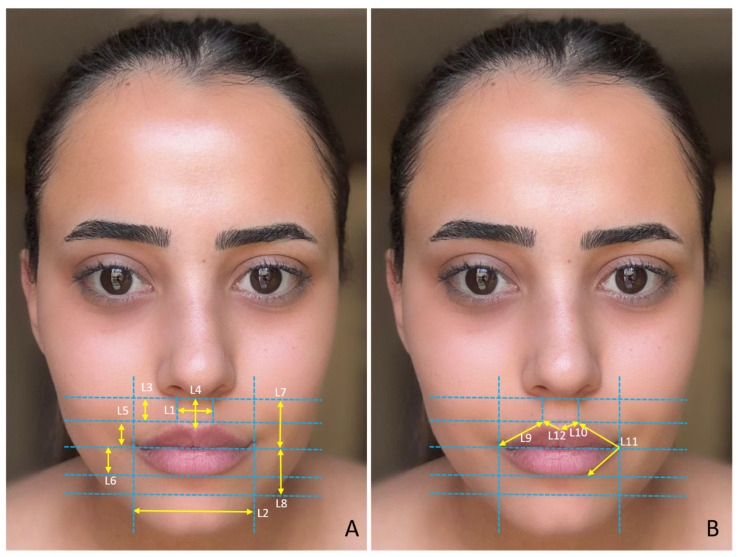
(**A**,**B**) Measured variables related to the lip. L1—philtrum width; L2—mouth width; L3—distance between crista philtrum and subalare; L4—philtrum height; L5—upper vermilion height; L6—lower vermilion height; L7—upper lip height; L8—lower lip height; L9—distance from chelion to crista philtrum; L10—distance between crista philtrum and stomion; L11—chelion angle; and L12—philtrum base angle.

The study was approved by the Harran University Faculty of Medicine Clinical Research Ethics Committee with the number HRU/21.19.15. To determine the sample size, we conducted a power analysis test based on the correlation between the face and facial organs using the G*Power ver. 3.1.9.7 (Germany) program. Using a two-sided test, α = 0.05, 80% power, and an effect size of 0.6, we calculated the required sample size as 98 *(n* = 98), 49 males and 49 females. In total, 100 volunteers, 50 male (mean age = 21.48 ± 1.54) and 50 female (mean age = 21.26 ± 0.66), participated in the study. Individuals with any congenital anomaly in the face and facial organs, those who had undergone surgical operations on the face and facial organs, those who had undergone cosmetic interventions (botox, facelift, fillers, etc.), those who were undergoing treatment such as hormone therapy, and those who were exposed to hair loss were not included in the study. We took facial photographs of the participants so that measurements of the face and facial organs could be made in a digital environment. We took the photographs in a standard position, in an upright sitting position, and in the Frankfurt Horizontal plane. The volunteers were instructed to refrain from making any facial movements or expressions during the photographs to ensure a neutral appearance. The same researcher took all the photographs and the same researcher made the measurements. The measured variables related to the face are shown in Figure 1A,B, the measured variables related to the eye are shown in Figure 2A,B, the measured variables related to the nose are shown in Figure 3A,B, and the measured variables related to the lip are shown in Figure 4A,B. We used Image J software Ver. 1.51 (Maryland, ABD) for the measurements.

### Statistical Analysis

Statistical analysis of the data was performed using SPSS Ver. 28.0 (IBM; New York, NY, USA) We used the Shapiro–Wilk test to test the normality of the data. For the comparison of the female and male groups, we used the independent sample *t* test (Student’s *t*-test) for normally distributed variables and the Mann–Whitney-U test for non-normally distributed variables. We performed correlation analysis to examine the relationship between variables. We developed linear regression equations to predict the measurements required for the most compatible morphometric features of the face and facial organs. We accepted the significance level as *p* < 0.05 in all our statistical analyses.

## 3. Results

Fifty male volunteers with a mean age of 21.48 ± 1.54 years and fifty female volunteers with a mean age of 21.26 ± 0.66 years participated in the study. According to the results of the Shapiro–Wilk normality test, the independent sample T test (Student’s *t*-test) was applied for variables with a normal distribution and the Mann–Whitney U test was applied for variables that were not normally distributed. As a result of the comparative statistical analysis, a statistically significant difference was found between men and women for the variables F5—lower face height, E5—palpebral fissure height, E6—distance between the margin of the upper eyelid and the eyebrow, E8—distance between the midpoint of the eye and the edge of the lower eyelid, N3—alar width, and N5—nasal root angle (Table 1).

As a result of the correlation analyses, linear regression equations were developed to estimate the ideal natural facial morphometry of men and women by the means of variables that were highly correlated with each other. The linear regression equations developed to estimate the general facial morphometric features of men and women are given in Table 2. According to these results, the variables with the highest estimation power and the lowest standard error of the estimate (SEE) were F6—inter-tragus width (adjusted R^2^ = 0.945; SEE = 2.66) for men and F2—upper face width (adjusted R^2^ = 0.958; SEE = 3.23) for women.

The linear regression equations developed for estimating the morphometric features of the eyes of males and females are given in Table 3. According to these results, the variables with the highest estimation power and the lowest standard deviation were E2—interpupillary distance (adjusted R^2^ = 0.934; SEE = 1.56) for men and E3—external intercantal distance (adjusted R^2^ = 0.954; SEE = 2.17) for women.

The linear regression equations developed for the estimation of the morphometric features of the noses of males and females are given in Table 4. According to these results, the variables with the highest estimation power and the lowest standard error of the estimate were N7—nasal height for men (adjusted R^2^ = 0.863; SEE = 2.11) and N3—alar width for women (adjusted R^2^ = 0.891; SEE = 1.43).

The linear regression equations developed for the prediction of morphometric features of the lips of men and women are given in Table 5. According to these results, the variables with the highest estimation power and the lowest standard error of the estimate were L7—upper lip height (adjusted R^2^ = 0.809; SEE = 1.69) for men and L6—lower vermilion height (adjusted R^2^ = 0.789; SEE = 0.85) for women.

## 4. Discussion

In our study, we addressed the harmony and balance of the morphometry of one’s own face and facial organs against the lack of a common consensus on the perception of beauty. While each population or gender may have its own unique characteristics, there are a balance and harmony that are appropriate for each individual’s own facial morphometry. Therefore, we developed equations to predict the optimal morphometric values based on individual characteristics. With the linear regression equations we developed, we aimed to predict the morphometric characteristics of the anatomically optimal structures that are harmoniously compatible with an individual’s face and facial features. For example, a measurement such as N7—nasal height, which is expected to be in balance and harmony with the overall facial contour and other facial features, can be estimated by inputting the relevant facial and facial organ measurements into the regression equation.

The face is the most important person-specific element in defining the physical image that can form an individual’s biological self-image. The interest in cosmetic treatment, such as aesthetic dental and smile designs, is increasing in terms of individuals’ belonging to a social community, job acceptance, social interactions, self-perception, and self-confidence [24,25,26].

Humans have long tried to characterize beauty on the basis of various rules and canons and have expressed the perception of beauty as symmetry, balance, and harmony [9]. Harmony, which means an optimal proportional relationship, is also used in craniofacial collocation [5].

Data on the anthropometric craniofacial parameters of the face and facial organs can serve clinical applications such as reconstructive and plastic surgery, maxillofacial surgery, prosthodontics, and orthodontics, as well as contributing to forensic applications [7,17,27].

According to Farkas, beauty is not an abstraction created from the artist’s imagination, but a biometric correlation and a tangible reality as long as it is based on measurable traits shown in somatic characters [21].

The anatomical structure of the face, the shape of the eyes and lips, and the smile are features that contribute to the attractiveness of a face as perceived by others. Due to the influence of facial physical features on attractiveness, the concept that “beauty is in the eye of the beholder” is often explored, and a more global perception of attractiveness is discussed [28]. However, not much focus is placed on self-perceived facial attractiveness, which has a strong link to self-esteem and self-confidence [29,30,31]. Facial attractiveness, which is desired by most members of society, is important in terms of being considered beautiful and more broadly “better” and receiving a wide range of social responses [32,33,34]. Jefferson stated that there is a close relationship between health and beauty and that emotional and psychological problems come with facial features perceived as unattractive [35].

According to Jefferson, there is a universal standard for facial beauty independent of race, age, gender, and other variables, and this universal standard is based on the divine proportion. The divine proportion is synonymous with beauty. Divinely proportioned faces and bodies are aesthetically attractive, healthy, fertile, and physiologically resilient [35]. However, according to Saeed et al., most explanations of the concept of beauty discussed by philosophers and psychologists are subjective, lacking scalability, generality, and accuracy. Furthermore, the diversity of age, gender, and ethnicity should be taken into account regarding the perception of beauty [36].

Multidisciplinary coordination is required to develop a smile that is compatible with the components of the face, teeth, lips, pupils, and nose, especially for orthodontic applications aiming to achieve perfect facial aesthetics. In some population-based studies, the relationship between the golden ratio and facial evaluation scores did not reach statistical significance. Therefore, in cases where the golden ratio does not need to be calculated, the calculation of a mathematical ratio would be helpful in diagnosis and treatment planning to improve a patient’s smile [26,37,38]. Therefore, in our study, we examined the mathematical harmony and order between the general anatomical structure of the face and the facial components of the eyes, nose, and lips to achieve an optimally attractive face.

For ophthalmologists and plastic surgeons, periocular morphologic assessment is important in periocular surgery because of its small area, elaborate structures, inadequate landmarks, and sensitive protective properties [39,40]. Measurements of anatomical structures are needed to monitor morphologic growth due to aging, diagnose malformations, plan surgical procedures, and evaluate surgical procedures or treatment outcomes [40,41]. In our study, regression equations were developed to predict the morphometric characteristics of the periorbital region based on the balance and harmony of a person’s face and facial organs (Table 3).

In rhinoplasty, which is one of the application areas of cosmetic surgery, a combination of aesthetic harmony with the surrounding facial features should be achieved while maintaining or establishing nasal function and support. The surgical procedure uses facial surface anthropometric features and measurements to analyze patients’ faces for surgeries, including rhinoplasty [42]. However, in rhinoplasty, the aesthetic features of the face and nose are often determined purely subjectively rather than through an objective assessment based on anthropometric proportional analysis. This is due to the lack of validated and reliable methods for measuring facial aesthetics and accurate nasal treatment planning [43,44,45]. Sak et al. stated that the soft tissue reconstruction of the nose should be conducted by taking into account the bone skeleton and linear regression equations developed for the construction of the most appropriate nasal shape using measurements made on bone structures [46]. In our study, we developed regression equations to predict the morphometric characteristics of the nose to be reconstructed in a way that best fits an individual’s own face and facial organs (Table 4).

The lips, which are at the center of lower facial aesthetics and are necessary to convey facial expressions and emotions, an integral part of daily life, play an important role in determining the overall aesthetic perception of the face. For this reason, cosmetic surgery as a means to modify the lips has recently gained popularity. However, there is no universally ideal lip that can be applied to a patient, so the patient’s age, ethnicity, sociocultural environment, and personal preference should be taken into account [47]. Babacan et al. stated that the shape of facial organs such as lips is related to the bony skeletal structure of an individual and that this feature should also be taken into account for facial reconstruction. In their study, they developed linear regression equations that predict the morphometric properties of the most appropriate lip shape for the bone structure of an individual using measurements taken from bone tissue [48]. Therefore, in our study, instead of a universal standard lip, we developed regression equations to predict the morphometric characteristics of the lips that best fit the morphometry of an individual’s own face and facial organs, taking into account soft tissue harmony and balance (Table 5).

## 5. Conclusions

We present a novel perspective on the field of facial beauty, which has attracted the attention of humanity for centuries and has recently received more attention. Facial beauty should not be associated with any standard, and the basis of facial beauty is the harmony and balance between the overall shape of the face and the facial organs. Harmony and balance vary according to population and gender norms. Recent cosmetic/aesthetic procedures generally follow standardized trends. However, a standardized procedure can disrupt the harmony and balance between an individual’s face or facial organs. This goes beyond the borders of ideal natural beauty. Therefore, in our study, we developed linear regression formulas to estimate the ideal natural optimum morphometric values, taking into account the population and gender of an individual. The equations developed will estimate the optimum morphometric features of a person for a natural harmony and balance between the face and facial organs in accordance with each individual’s population and gender. Comparative statistical analyses revealed genetically and hormonally based differences between male and female facial and facial organ morphometry. Accordingly, we developed sex-specific regression equations for both males and females. Therefore, the application of these equations in clinical practice should be approached with the consideration of the individual’s sex to ensure accuracy and relevance. We believe that our study will guide medical professionals who perform cosmetic/aesthetic interventions and also inspire software or artificial intelligence applications related to facial or facial organ design.

## 6. Limitations

***Limited Sample Diversity:***

The study sample consisted of young adult volunteers with a narrow age range (mean age = 21.48 ± 1.54) and 50 females (mean age = 21.26 ± 0.66), limiting the generalizability of the findings to other age groups, such as children or older adults. Broader age-related morphometric variations could not be assessed.

***Population and Cultural Homogeneity:***

All participants were from the same population and cultural background. Since perceptions of beauty and facial proportions vary significantly across populations, the regression equations may not be directly applicable to individuals from other population groups.

***Two-Dimensional Imaging Constraints:***

Facial measurements were obtained from two-dimensional digital photographs. This method does not capture depth-related parameters, which can be important in a comprehensive assessment of facial morphology.

***Facial Expressions and Muscle Tone:***

Although participants were instructed to maintain a neutral facial expression, subtle involuntary muscle tone variations may have influenced certain measurements, especially around dynamic areas like the eyes and lips.

***Exclusion of General Body Metrics:***

The study focused solely on the face and facial organs without considering general body metrics such as Body Mass Index (BMI), which may influence facial soft tissue distribution and, thus, affect morphometry.

***Cross-Sectional Design:***

The cross-sectional nature of the study does not allow for tracking facial morphometric changes over time or assessing how aging might alter facial harmony and proportions.

***Absence of Functional or Subjective Aesthetic Evaluation:***

The study focused on anatomical proportions and did not incorporate subjective beauty ratings or functional assessments (e.g., breathing for nasal structure), which could have provided a more comprehensive analysis.

## Figures and Tables

**Table 1:** Comparative statistical analysis results between men and women.

Variables	Gender	Med (Min-Max)	Mean ± SD	*p*
F5—lower face height (distance between nasion and menton)	M (*n* = 50)	55.59 (41.79–120.79)	60.39 ± 13 43	<0.001
	Fm (*n* = 50)	52.27 (33.63–68.52)	52.26 ± 7.84	
E5—palpebral fissure height (distance between upper and lower eyelids)	M (*n* = 50)	10.21 (5.98–14.99)	10.12 ± 1.96	<0.001
	Fm *(n* = 50)	12.65 (7.91–29.11)	12.78 ± 3.76	
E6—distance between the margin of the upper eyelid and the eyebrow	M (*n* = 50)	7.08 (3.36–11.33)	6.96 ± 1.78	<0.001
	Fm (*n* = 50)	8.91 (3.96–17.21)	8.81 ± 2.46	
E8—distance between the midpoint of the eye and the edge of the lower eyelid	M (*n* = 50)	4.07 (2.08–7.37)	4.19 ± 1.23	0.001
	Fm (*n* = 50)	3.99 (2.38–5.51)	4.03 ± 0.72	
N3—alar width	M (*n* = 50)	38.90 (32.90–52.59)	39.40 ± 4.62	0.006
	Fm (*n* = 50)	36.53 (26.69–44.38)	36.27 ± 4.34	
N5—nasal root angle	M (*n* = 50)	47.02 (34.22–57.73)	47.01 ± 4.94	0.031
	Fm (*n* = 50)	42.98 (33.47–53.59)	42.88 ± 3.63	

M—Male; Fm—Female; Med—Median; Min—Minimum; Max—Maximum; SD—Standard Deviation.

**Table 2:** Linear regression equations to estimate the morphometric features of facial variables.

Variable	Gender	Equation	Adjusted R^2^	SEE
F1—facial height	M (*n* = 50)	F1 = 13.843 + (0.766 × L7) + (0.788 × L8) + (0.781 × L3) + (0.315 × F3) + (0.475 × F4) + (0.190 × E11) − (0.271 × E12)	0.911	3.42
	Fm (*n* = 50)	F1 = 23.750 + (1.060 × F5) + (0.522 × F4) − (0.480 × N5) + (0.544 × N3)	0.943	3.23
F2—upper face width	M (*n* = 50)	F2 = −7.233 + (1.048 × F6) − (0.508 × F7) + (0.965 × L7) + (0.379 × L2) − (1.367 × E8)	0.901	4.13
	Fm (*n* = 50)	F2 = −1.253 + (0.475 × F6) + (0.897 × E1) + (0.291 × L2) + (0.165 × F3) − (0.173 × E4)	0.958	3.23
F3—lower face width	M (*n* = 50)	F3 = 16.869 + (0.936 × F6) − (0.152 × L12) − (1.449 × E5) + (0.204 × E11)	0.804	4.84
	Fm (*n* = 50)	F3 = 9.542 + (0.512 × F2) − (0.804 × E6) + (0.350 × F6)	0.815	4.97
F4—upper face height	M (*n* = 50)	F4 = 22.465 + (0.088 × F8) − (0.543 × N5) + (0.619 × N2) + (0.200 × N7) + (0.358 × L9) + (0.649 × E9)	0.833	2.56
	Fm (*n* = 50)	F4 = 21.229 + (0.087 × F8) + (0.370 × N4) + (0.327 × E1) − (0.434 × N5) + (0.585 × N3)	0.848	2.76
F5—lower face height	M (*n* = 50)	F5 = 28.721 + (0.992 × F1) + (0.276 × F8) + (1.715 × E5) − (4.959 × E9) + (1.044 × N4) − (0.817 × N6) − (1.630 × L2)	0.450	9.97
	Fm (*n* = 50)	F5 = −15.347 + (0.573 × F1) − (0.562 × F4) + (0.147 × F8) + (0.268 × N5)	0.909	2.36
F6—inter-tragus width	M (*n* = 50)	F6 = 5.910 + (0.158 × E3) + (0.300 × F3) + (0.361 × F2) + (0.308 × F7)	0.945	2.66
	Fm (*n* = 50)	F6 = 12.767 + (0.635 × F2) + (0.473 × F7) − (1.028 × E1) + (0.708 × E2) − (0.589 × L9) − (0.362 × N8) + (0.632 × L5)	0.957	2.97
F7—facial width	M (*n* = 50)	F7 = −4.708 + (0.478 × E3) + (0.492 × L2) − (1.396 × E10) + (0.737 × F6) − (0.557 × F2) + (0.531 × N3)	0.849	3.94
	Fm (*n* = 50)	F7 = −17.504 + (0.613 × F6) + (0.726 × L9) + (0.409 × N8) + (0.399 × E1)	0.931	3.29
F8—total facial height	M (*n* = 50)	10.904 + (0.529 × F1) + (0.525 × F6) + (1.817 × E9) + (0.518 × F4)	0.835	7.01
	Fm (*n* = 50)	F8 = −6.077 + (0.602 × F1) + (1.066 × E3) + (0.983 × N4)	0.897	6.90

**Table 3:** Linear regression equations to estimate the morphometric features of eye variables.

Variable	Gender	Equation	Adjusted R^2^	SEE
E1—internal intercantal distance	M (*n* = 50)	E1 = −4.70 + (0.052 × F5) − (0.052 × F8) + (0.499 × E3) − (0.632 × E5) + (0.451 × E8) − (0.174 × N5) + (0.183 × L2) + (0.791 × L5) − (0.302 × L6) − (0.211 × L7)	0.706	2.13
	Fm (*n* = 50)	E1 = −4.660 + (0.236 × E3) − (0.237 × F6) + (0.206 × F2) + (0.313 × E2)	0.868	1.68
E2—interpupillary distance	M (*n* = 50)	E2 = 4.648 − (0.457 × L5) + 0.516 ×E3) + (0.929 × L6) + (0.887 × E7) − (0.134 × N6) − (0.560 × L5) + (0.227 × E1)	0.934	1.56
	Fm (*n* = 50)	E2 = −1.078 + (0.513 × E3) + (0.257 × N1) + (0.117 × F7)	0.950	1.62
E3—external intercantal distance	M (*n* = 50)	E3 = 5.8959 + (0.944 + E2) + (0.171 × F7) + (0.214 × N6) + (0.344 × N1) − (0.662 × L6)	0.933	2.18
	Fm (*n* = 50)	E3 = 8.754 + (0.821 × E2) + (0.113 × F8) + (0.423 × E1)	0.954	2.17
E4—palpebral fissure length	M (*n* = 50)	E4 = 0.817 − (0.067 × F3) + (0.194 × F6) − (0.580 × E7) + (0.114 × E11) − (0.109 × E12) + (0.305 × L3) + (0.186 × E2)	0.711	1.81
	Fm (*n* = 50)	E4 = 5.260 + (0.117 × E3) − (0.937 × E5) + (1.137 × E7) + (1.661 × E8) + (0.107 × F1)	0.848	2.13
E5—palpebral fissure height	M (*n* = 50)	E5 = −1.307 − (0.062 × F3) + (0.042 × F5) − (0.120 × E1) + (0.084 × E3) + (0.204 × E6) + (0.745 × E7) + (0344 × E8) + (0.056 × E11) + (0.184 × N2) − (0.201 × N3) + (0.092 × L2) − (0.183 × L6)	0.798	0.88
	Fm (*n* = 50)	E5 = 7.687 − (0.694 × E4) + (0.169 × F6) + (1.223 × E7) − (0.285 × L1)	0.848	2.13
E6—distance between the margin of the upper eyelid and the eyebrow	M (*n* = 50)	E6 = 2.314 + (0.033 × E2) + (0.351 × E5) − (0.367 × E7) + (0.736 × E9) − (0.708 × E10) + (0.168 × L8)	0.426	1.35
	Fm (*n* = 50)	E6 = −10.910 + (0.254 × N3) + (0.301 × L4) + (0.054 × L12)	0.507	1.73
E7—distance between the midpoint of the eye and the edge of the upper eyelid	M (*n* = 50)	E7 = −2.283 − (0.031 × F1) + (0.219 × E2) − (0.122 × E3) + (0.503 × E5) − (0.279 × E8) + (0.206 × N3) − (0.110 × N4)	0.752	0.85
	Fm (*n* = 50)	E7 = −2.606 + (0.050 × F1) + (0.194 × L8) + (0.039 × E11)	0.579	0.95
E8—distance between the midpoint of the eye and the edge of the lower eyelid	M (*n* = 50)	E8 = −6.366 − (0.081 × F2) + (0.025 × F3) + (0.311 × E9) + (0.040 × E12) + (0.110 × N3) (0.077 × N7) + (0.027 × L12)	0.537	0.83
	Fm (*n* = 50)	E8 = 0.952 + (0.138 × E4) − (0.108 × L7) + (0.091 × E5)	0.427	0.54
E9—vertical diameter of the iris (apparent)	M (*n* = 50)	E9 = 2.769 + (0.092 × F1) − (0.045 × F3) + (0.177 × E6) + (0.633 × E7) + (0.480 × E8) + (0.025 × E11) − (0.157 × N3) + (0.207 × N4) − (0.080 × N6) − (0.270 × L5)	0.704	0.88
	Fm (*n* = 50)	E9 = −4.239 + (0.066 × E3) + (0.060 × L11) + (0.035 × E11) + (0.328 × E10)	0.724	0.82
E10—horizontal diameter of the iris	M (*n* = 50)	E10 = 0.613 + (0.113 × L7) + (0.389 × E9) + (0.126 × E4) − (0.150 × E6) + (0.040 × N5)	0.769	0.64
	Fm (*n* = 50)	E10 = −0.012 + (0.080 × E2) + (0.270 × L10) + (0.258 × L3) − (0.166 × N8) + (0.099 × N2) + (0.176 × N7) + (0.323 × E9) − (0.135 × N1) − (0.058 × E4) − (0.0856 × N6)	0.904	0.47
E11—lateral canthus angle	M (*n* = 50)	E11 = 10.524 + (1.876 × E5) + (0.499 × E12) + (1.382 × E9) − (1.119 × N2) + (0.701 × E1)	0.620	5.63
	Fm (*n* = 50)	E11 = 43.824 + (0.594 × E12) + (0.646 × E9) − (0.802 × N2) − (0.553 × N5)	0.382	6.32
E12—medial canthus angle	M (*n* = 50)	E12 = 29.096 − (0.382 × F1) − (0.786 × E4) + (1.501 × N2) − (0.544 × N4) + (1.153 × L3) − (0.516 × L4) + (0.551 × L8) + (0.624 × E11)	0.512	5.27
	Fm (*n* = 50)	E12 = 51.127 − (0.699 × E2) + (1.882 × E10) + (0.711 × N3) − (0.444 × N7) + (0.783 × N8) − (0.663 × L3) − (1.887 × L5) + (1.519 × L6)	0.206	5.21

**Table 4:** Linear regression equations to estimate the morphometric features of nasal variables.

Variable	Gender	Equation	Adjusted R^2^	SEE
N1—nasal root width	M (*n* = 50)	N1 = −0.597 + (0.049 × F1) + (0.244 × E3) + (0.456 × E7) − (0.134 × F4) − (0.436 × E9) − (0.070 × N5) + (0.116 × L6)	0.562	1.78
	Fm (*n* = 50)	N1 = 0.903 + (0.325 × E1) + (0.871 × E9) − (1.208 × E10) + (0.326 × N2) − (0.088 × N8) + (0.379 × L3) − (0.313 × L8) + (0.628 × L10)	0.583	1.93
N2—nasal dorsum width	M (*n* = 50)	B2 = 4.208 + (0.089 × F7) + (0.265 × E4) + (0.555 × E5) − (0.189 × E11) + (0.162 × E12) + (0.387 × N4) − (0.127 × N6) − (0.367 × L3) + (0.308 × L4) + (0.265 × L7)	0.837	1.39
	Fm (*n* = 50)	N2 = −0.451 + (0.130 × F3) + (0.356 × N1) + (0.280 × N4) + (0.156 × L2) − (0.179 × L9)	0.638	2.39
N3—alar width	M (*n* = 50)	N3 = −13.932 + (0.318 × N4) + (0.291 × N5) + (0.169 × F1) + (0.767 × E7) + (0.089 × F7)	0.835	1.87
	Fm (*n* = 50)	N3 = −6.230 + (0.264 × N2) + (0.283 × N5) + (0.224 × F4) + (0.351 × L6) + (0.311 × E6) + (01.71 × L2) − (0.023 × L12)	0.891	1.43
N4—subalar width	M (*n* = 50)	N4 = 0.212 − (0.137 × F1) − (0.482 × E7) + (0.119 × E11) − (0.182 × E12) + (0.541 × N2) + (0.410 × N3) + (0.177 × N6)	0.639	1.80
	Fm (*n* = 50)	N4 = −14.778 + (0.200 × N2) + (0.155 × N5) + (0.274 × F4) + (0.020 × L12) − (0.241 × E6) + (10.129 × L2)	0.478	2.71
N5—nasal root angle	M (*n* = 50)	N5 = 45.234 − (0.615 × F4) + (0.174 × F7) + (1.578 × E10) − (0.188 × E11) + (0.455 × N3) − (0.853 × N3) + (0.341 × L4)	0.730	2.56
	Fm (*n* = 50)	N5 = 41.399 − (0.589 × F4) + (0.650 × E10) + (0.814 × N3) − (0.196 × L4)	0.360	2.91
N6—nasal length	M (*n* = 50)	N6 = 15.461 + (0.709 × N7) + (0.795 × L3) − (0.973 × L1) − (0.304 × N5) − (1.957 × E7) + (0.211 × F2 + (0.758 × L5)	0.823	2.80
	Fm (*n* = 50)	N6 = 2.957 + (0.935 × N7) − (0.194 × E4) + (0.423 × L6)	0.835	2.14
N7—nasal height	M (*n* = 50)	N7 = 0.863 − (0.643 × E6) + (1.064 × E8) + (0.872 × E9) + (0.934 × N8) + (0.609 × L1) − (0.249 × L7) − (0.749 × L10) + (0.412 × N6)	0.863	2.11
	Fm (*n* = 50)	N7 = −0.470 + (0.705 × N6) + (0.398 × N8) + (0.836 × E10) − (0.246 × L7)	0.867	1.88
N8—nasal tip projection	M (*n* = 50)	N8 = 13.328 + (0.054 × F1) − (0.373 × E9) + (0.362 × E10) + (0.380 × N7) − (0.276 × L1) − (0.125 × L2) + (0.377 × L10)	0.705	1.43
	Fm (*n* = 50)	N8 = 1.171 + (0.586 × N7) + (0.817 × L3) − (1.868 × E10) + (0.476 × N2) + (0.790 × L10) − (0.430 × N3) + (0.754 × E9)	0.714	1.92

**Table 5:** Linear regression equations to estimate the morphometric features of lip variables.

Variable	Gender	Equation	Adjusted R^2^	SEE
L1—philtrum width	M (*n* = 50)	L1 = −0.708 + (0.084 × F8) + (0.513 × E5) − (0.851 × E7) − (0.930 × E8) − (0.092 × E11) + (0.078 × E12) − (0.212 × N6) + (0.168 × N7) + (0.835 × L10)	0.545	1.66
	Fm (*n* = 50)	L1 = −3.062 + (0.227 × F2) − (0.105 × F6) − (0.151 × E1) + (0.172 × N3) + (0.129 × N4) − (0.121 × L9) + (0.366 × L10)	0.523	1.75
L2—mouth width	M (*n* = 50)	L2 = 0.961 + (0.216 × F3) − (0.075 × F5) − (0.251 × F6) + (0.258 × F7) −(0.831 × E8) + (0.563 × N3) − (0.229 × N5) + (0.442 × L6) + (0.098 × L12)	0.719	2.79
	Fm (*n* = 50)	L2 = 18.385 + (0.142 × F8) − (0.177 × L11) + (0.436 × N3)	0.762	2.53
L3—distance between crista philtrum and subalare	M (*n* = 50)	L3 = −2.192 + (0.693 × L4) + (0.297 × E2) − (0.267 × N2) − (0.058 × E11)	0.801	1.30
	Fm (*n* = 50)	L3 = 7.205 + (0.332 × L7) + (0.275 × L4) − (0.082 × L11)	0.665	1.32
L4—philtrum height	M (*n* = 50)	L4 = 2.221 + (1.061 × L3) − (0.356 × E2) + (0.450 × N2) + (0.083 × E11)	0.785	1.61
	Fm (*n* = 50)	L4 = −5.927 + (0.650 × L3) 0 (0.244 × F5) + (0.456 × E6) − (0.086 × F6) + (0.071 × E6)	0.696	1.91
L5—upper vermilion height	M (*n* = 50)	L5 = 1.899 + (0.451 × L6) + (0.218 × L9) − (0.073 × N7) + (0.290 × E5) − (0.228 × E2) + (0.171 × E1) + (0.066 × L11) + (0.227 × L10) + (0.238 × E7)	0.784	0.80
	Fm (*n* = 50)	L5 = −0.763 − (0.122 × F4) + (0.031 × F8) + (0.121 × E6) − (0.124 × L4) + (0.184 × L7) + (0.428 × L8)	0.616	1.16
L6—lower vermilion height	M (*n* = 50)	L6 = −0.739 + (0.606 × L5) + (0.162 × L7) − (0.422 × E5) + (0.078 × N7) + (0.100 × L8)	0.532	1.13
	Fm (*n* = 50)	L6 = −2.922 + (0.462 × L8) + (0.068 × L11) − (0.163 × L4) + (0.042 × F1)	0.789	0.85
L7—upper lip height	M (*n* = 50)	L7 = −13.444 + (0.149 × F2) + (0.445 × L5) + (0.358 × L3) + (0.261 × N2) + (0.089 × L11)	0.809	1.69
	Fm (*n* = 50)	L7 = −10.321 + (0.117 × F1) + (0.705 × L3) + (0.431 × L5) + (0.053 × L12)	0.759	1.83
L8—lower lip height	M (*n* = 50)	L8 = 1.337 + (0.312 × F1) − (0.236 × F4) + (0.526 × E6) − (0.057 × L12) + (0.812 × L6) − (0.466 × L7)	0.562	2.36
	Fm (*n* = 50)	L8 = 0.583 + (0.004 × F1) − (0.012 × E6) + (0.953 × L6) + (0.201 × L7)	0.743	1.32
L9—distance from chelion to crista philtrum	M (*n* = 50)	L9 = 13.465 + (0.856 × L5) + (0.134 × F4) − (0.136 × L11) − (0.340 × L4) + (0.381 × L7) − (0.497 × L6)	0.586	1.82
	Fm (*n* = 50)	L9 = 10.188 + (0.164 × F7) − (0.254 × E5) − (0.148 × L11) + (0.336 × L8)	0.581	2.05
L10—distance between crista philtrum and stomion	M (*n* = 50)	L10 = −4.944 − (0.050 × F3) + (0.068 × F6) − (0.026 × F8) − (0.145 × E5) + (0.413 × E8) + (0.060 × E11) − (0.206 × N7) + (0.345 × N8) + (0.348 × L1) + (0.106 × L2)	0.533	1.08
	Fm (*n* = 50)	L10 = 2.181 + (0.267 × E7) − (0.335 × E9) + (0.858 × E10) − (0.107 × N2) − (0.161 × N7) + (0.201 × N8) + (0.097 × L2) − (0.456 × L3) + (0.138 × L4)	0.419	1.04
L11—chelion angle	M (*n* = 50)	L11 = 89.222 − (0.177 × F3) − (0.208 × F8) + (0.701 × E3) − (0.990 × N1) − (1.347 × L4) + (2.155 × L5) + (1.245 × L7) − (1.354 × L9) − (0.242 × L12)	0.640	4.64
	Fm (*n* = 50)	L11 = 36.533 + (2.135 × L6) − (0.772 × L9) + (2.806 × E9) − (0.474 × E5) − (0.324 × F4)	0.574	4.71
L12—philtrum base angle	M (*n* = 50)	L12 = 119.143 + (0.944 × F2) − (0.805 × F3) + (0.755 × E3) + (1.496 × E6) + (2.524 × E8) − (2.693 × E9) − (1.705 × N3) + (0.847 × N5) − (1.423 × L4) − (0.551 × L11)	0.451	9.20
	Fm (*n* = 50)	L12 = 152.268 − (0.340 × F3) + (0.636 × E3) + (1.957 × E6) − (4.901 × E8) − (1.002 × N3) − (1.393 × L4)	0.198	10.69

## Data Availability

The data presented in this study are available on request from the corresponding author.

## Abbreviations

The following abbreviations are used in this manuscript:

**F1**	Facial height (distance between nasion and menton)
**F2**	Upper face width (distance between external corner of the right eye and external corner of the left eye)
**F3**	Lower face width (bigonial width)
**F4**	Upper face height (distance between nasion and subnasale)
**F5**	Lower face height (distance between nasion and menton)
**F6**	Inter tragus width
**F7**	Facial width (bizygomatic width)
**F8**	Total facial height
**E1**	Internal intercantal distance
**E2**	Interpupillary distance
**E3**	External intercantal distance
**E4**	Palpebral fissure length (distance between medial and lateral canthus (eye width))
**E5**	Palpebral fissure height (distance between upper and lower eyelids)
**E6**	The distance between the margin of the upper eyelid and the eyebrow
**E7**	Distance between the midpoint of the eye and the edge of the upper eyelid
**E8**	Distance between the midpoint of the eye and the edge of the lower eyelid
**E9**	Vertical diameter of the iris (apparent)
**E10**	Horizontal diameter of the iris (apparent)
**E11**	Lateral canthus angle
**E12**	Medial canthus angle
**N1**	Nasal root width
**N2**	Nasal dorsum width
**N3**	Alar width
**N4**	Subalar width
**N5**	Nasal root angle
**N6**	Nasal length
**N7**	Nasal height
**N8**	Nasal tip projection
**L1**	Philtrum width
**L2**	Mouth width
**L3**	Distance between crista philtrum and subalare
**L4**	Philtrum height
**L5**	Upper vermilion height
**L6**	Lower vermilion height
**L7**	Upper lip height
**L8**	Lower lip height
**L9**	Distance from chelion to crista philtrum
**L10**	Distance between crista philtrum and stomion
**L11**	Chelion angle
**L12**	Philtrum base angle
